# P-731. Prevalence of Human Papillomavirus Infection in Males by Anatomical Site: A Worldwide Systematic review and 504Meta-Analysis

**DOI:** 10.1093/ofid/ofaf695.942

**Published:** 2026-01-11

**Authors:** Lara M Cruz, Alberto D’ávila, Andrea Ribeiro, Vera Saddi

**Affiliations:** Pontifícia Universidade Católica de Goiás, Goiânia, Goias, Brazil; Pontifícia Universidade Católica de Goiás, Goiânia, Goias, Brazil; Pontifícia Universidade Católica de Goiás, Goiânia, Goias, Brazil; Pontifícia Universidade Católica de Goiás, Goiânia, Goias, Brazil

## Abstract

**Background:**

Studies on HPV still focus mostly on women, leaving gaps regarding infection in men. This meta-analysis is the first to globally estimate HPV prevalence by anatomical site in men. Given the association with anal, penile, and oropharyngeal cancers, these findings address a critical issue and support more inclusive public health policies.

**Table 1.**
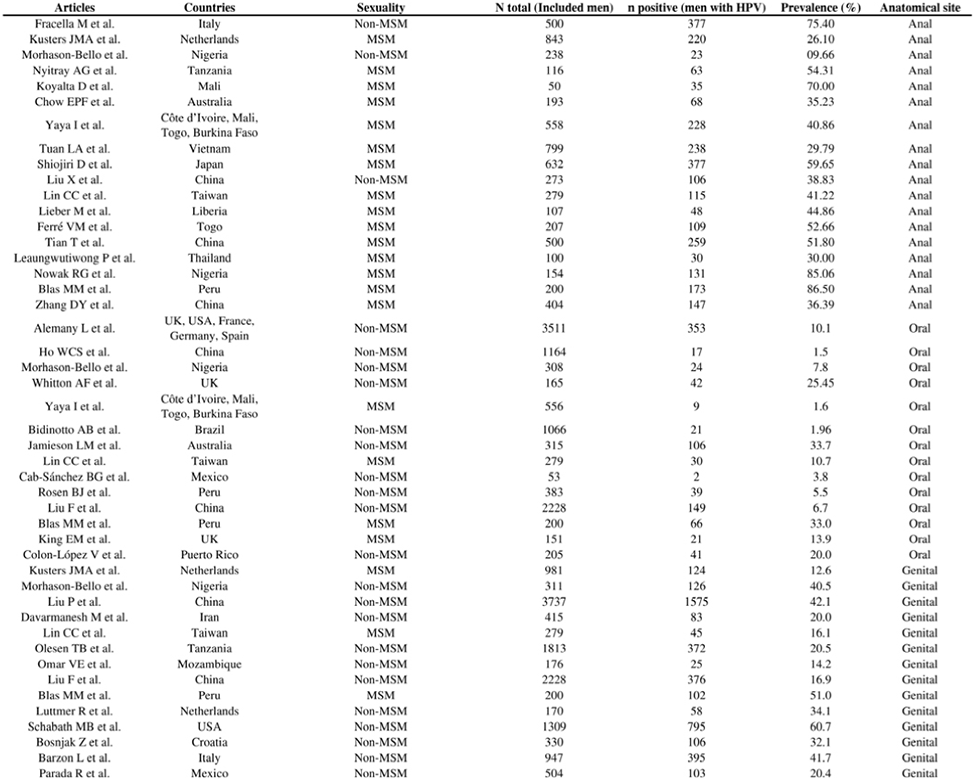
Characteristics of studies included by anatomical site, sexuality, and HPV prevalence. Abbreviations: MSM = Men who have sex with men

**Figure:**
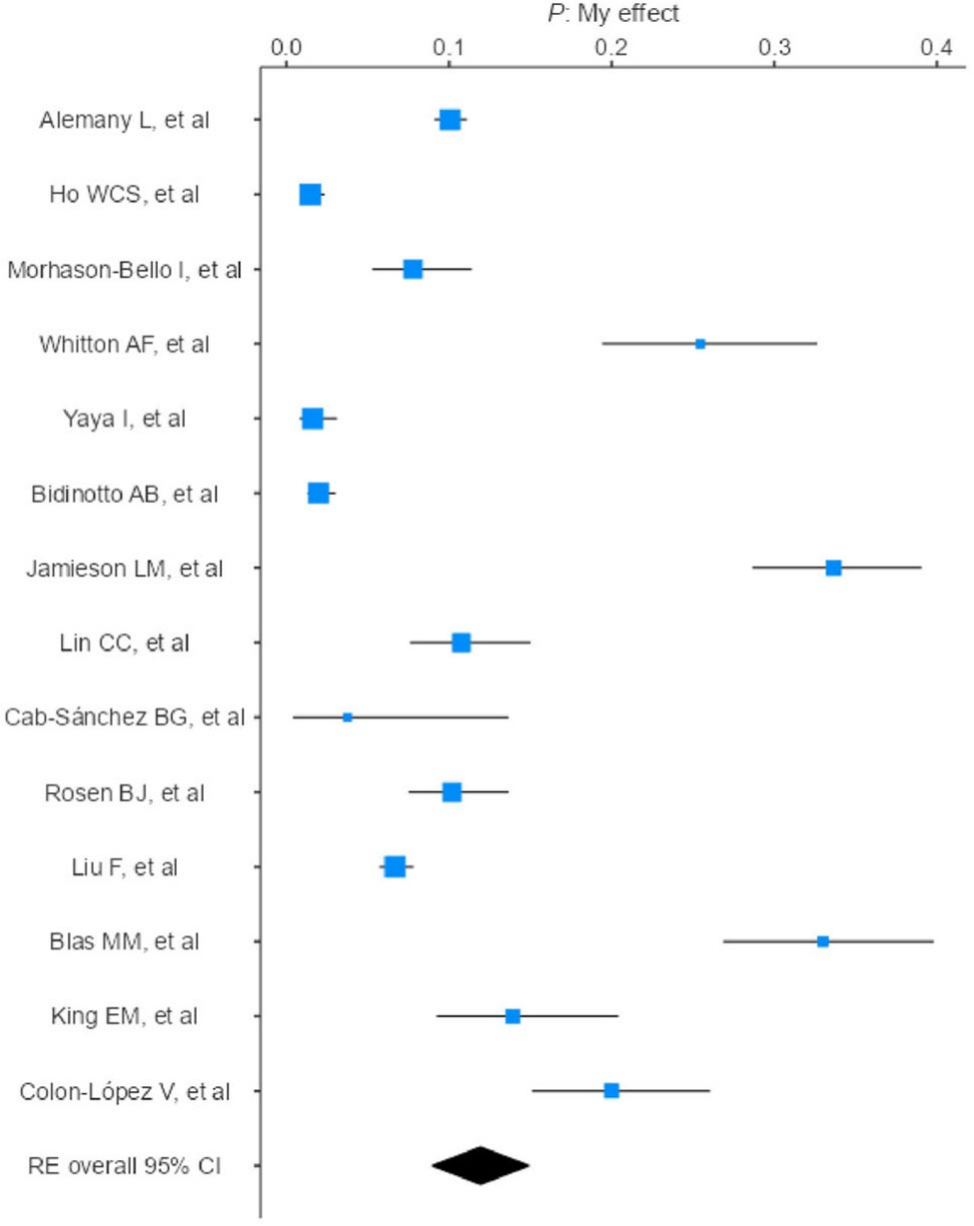
Forest plot - Oral HPV

**Methods:**

A meta-analysis was conducted according to PRISMA. PubMed and Embase were searched (2010–2025). The search strategy used descriptors such as “HPV”, “male” and “prevalence”, resulting in 269 studies screened. This review included observational studies reporting HPV prevalence in male populations, stratified by anatomical site (anal, genital, oral). Only studies using site-specific sampling were considered (swab, brush, oral rinse). Studies focused on genital warts, cancer, urine/semen samples, lack of diagnostic description, male-specific data, or limited to HIV-positive populations were excluded. Analyses and forest plots were made using Jamovi. The final sample included 27,350 men.

**Figure:**
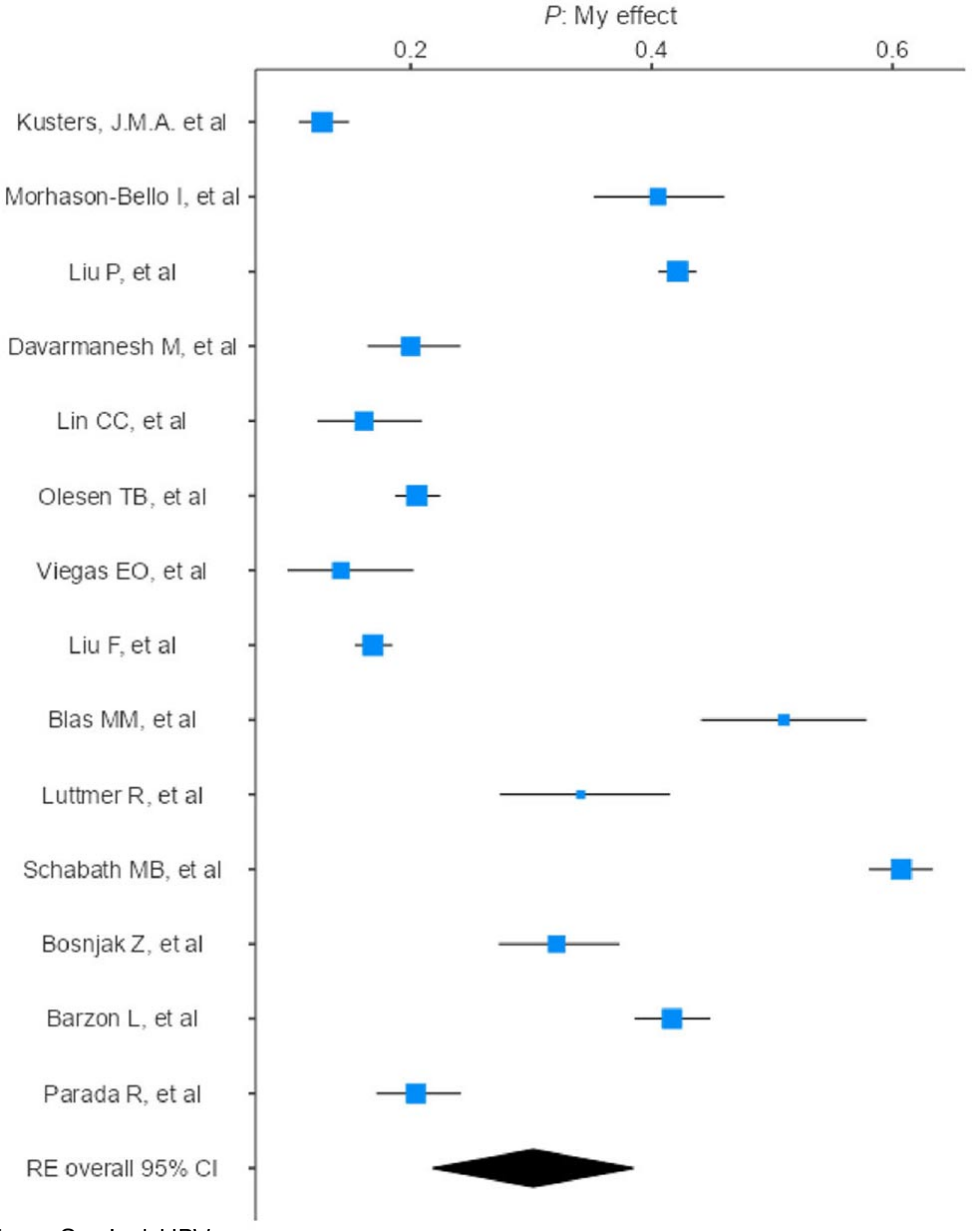
Forest plot - Genital HPV

**Figure:**
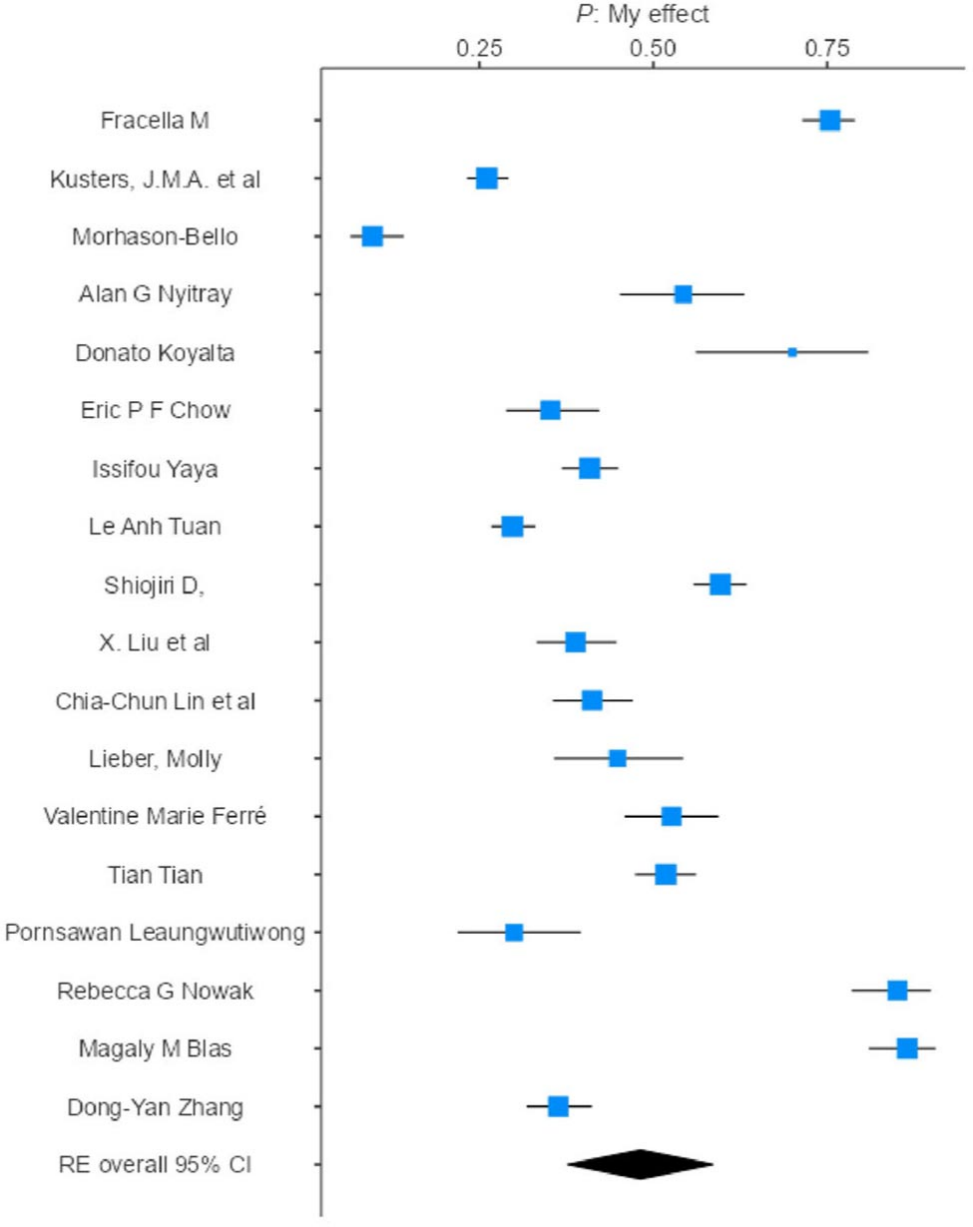
Forest plot - Anal HPV

**Results:**

Across 37 studies, including some contributing to multiple sites, overall HPV prevalence in anal, genital, and oral sites was 48.2% (95% CI: 37.7–58.6), 30.2% (95% CI: 21.8–38.5), and 11.9% (95% CI: 8.9–14.9), respectively. Among men who have sex with men (MSM), prevalence was 49.6%, 26.3%, and 13.7%, respectively. Among non-MSM, prevalence was 41.3%, 31.2%, and 11.4%, respectively. By region, anal HPV prevalence ranged from 40.4% (Asia-Pacific) to 50.8% (Africa); genital prevalence from 23.8% (Asia-Pacific) to 44.0% (Americas); and oral prevalence ranged from 4.6% (Africa) to 16.0% (Europe). No subgroup or regional difference was statistically significant.

**Conclusion:**

This global analysis revealed high HPV prevalence in men in all sites. Anal infections were more frequent in Europe (50.7%) and Africa (50.8%), while genital and oral infections were higher in the Americas and Europe. Although no significant differences between MSM and non-MSM were found, a higher trend among MSM was noted. High heterogeneity and scarce Latin American data showed important gaps. Expanding male vaccination is critical, as studies showed that vaccination in both genders reduced HPV infection rates in the overall population.

**Disclosures:**

All Authors: No reported disclosures

